# Can we optimise the TB response amid funding constraints using evidence-based, cost-effective strategies

**DOI:** 10.5588/ijtldopen.25.0771

**Published:** 2026-04-13

**Authors:** M.A. Yassin, A. Rashid, E. Wandwalo

**Affiliations:** 1 The Global Fund to Fight AIDS, Tuberculosis and Malaria, Geneva, Switzerland;; 2 Independent Consultant, Dar-es-Salaam, Tanzania.

**Keywords:** tuberculosis, funding cuts, innovations along TB care, Global Fund, digitalisation, innovative financing

## Abstract

**BACKGROUND:**

Despite progress in the TB response, including through the Global Fund’s catalytic efforts, funding gaps and inefficiencies threaten efforts to sustain gains and accelerate the TB response. Recent donor funding cuts have further strained programmes in low- and middle-income countries.

**METHODS:**

A mixed-method, combining desk and literature review and key informant interviews with national TB programme stakeholders, technical experts, and partners, was conducted using standardised tools. Findings were triangulated to identify strategies/approaches that improve efficiency, access, and outcomes.

**RESULTS:**

We identified cost-effective strategies that optimise resources and tools to sustain and expand access to services amid declining funding. Strategies to enhance efficiency include screening using digital X-rays (with artificial intelligence), testing samples (including pooled samples) with molecular diagnostics, decentralising care, engaging communities and private sector, and integrating TB services with broader health system. Cost-saving measures involve integrated sample transport and supervision, integrating TB with other diseases such as HIV, diabetes, and undernutrition, task shifting, shorter treatment regimens, digital tools, and e-learning. Emphasis was also placed on innovative financing, domestic resource mobilisation, and country ownership.

**CONCLUSION:**

Accelerating to end TB requires integrated and efficient strategies, innovative financing, and increased domestic resources, ensuring provision of equitable and high-quality TB services while strengthening the health system and ensuring sustainability.

TB remains a major global public health threat, with an estimated 10.7 million people with TB and 1.23 million deaths in 2024.^1^ The rise in TB incidence between 2021 and 2023 is largely attributed to the widespread service disruptions during COVID-19, which caused TB notifications to drop from 7.1 million in 2019 to 5.8 million in 2020. These declines contributed to a substantial increase in undiagnosed and untreated people with TB, thereby exacerbating transmission and increased incidence and mortality. However, in 2024, the highest ever number of people with TB were notified (8.3 million), signalling a full recovery and improvement in case finding.^2^

The Global Fund has been instrumental in advancing the global response to HIV, TB, and malaria, helping save approximately 70 million lives over the past two decades. Its strategic investments have catalysed innovation, fostered market shaping, and facilitated the adoption of cutting-edge technologies including artificial intelligence (AI)–enabled digital X-rays for screening, rapid molecular diagnostics, and shorter, more effective, patient-friendly treatment. These efforts have improved access, treatment coverage, and greater efficiency in TB care. Between 2002 and 2024, the Global Fund allocated over $11 billion to TB programmes in low- and middle-income countries (LMICs), helping reduce TB mortality by 40%. In 2024 alone, over 7.4 million people received TB treatment, including 120,000 treatment for drug-resistant TB (DR-TB), and 5.6 million received TB preventive treatment (TPT).^2^

Achieving the global targets of ending TB by 2030 requires increasing investments to $22 billion by 2027 and $35 billion by 2030 as committed at the 2023 United Nation High-Level Meeting.^3^ These investments could prevent up to one million deaths annually and yield $46 in return per $1 spent, driving major health and socio-economic gains.^4^ However, the global TB response remains severely underfunded, with only 27% ($5.9 billion) of the $22 billion available in 2024, with major gaps in critical health products in the Global Fund Grant Cycle 7 (2024–2026). Recent TB funding cuts and reductions have further disrupted programmes, especially in LMICs.^5^ In this context, enhancing programmatic efficiency is crucial to protect hard-won gains, maximise resources, sustain progress towards ending TB, and strengthen resilient and integrated health systems.

The aim of our study was to draw evidence on cost-effective, scalable TB interventions that optimise resources and tools to address funding gaps, inefficiencies, and inequities and offer key lessons to support sustainable and resilient global TB response.

## METHODS

This study employed a mixed-methods approach, combining review of the literature and reports with key informant interviews (KIIs). Guided by a health systems approach, it focused on structural, institutional, and individual factors influencing TB programmes efficiency and optimisation, amid funding constraints. The literature and desk reviews were targeted and designed to synthesise the most relevant recent evidence for practical recommendations. These covered publications from 2019 to 2024, a period marked by significant shifts in TB funding, policy, and service delivery, particularly due to COVID-19 and changing donor landscapes and priorities. Sources included peer-reviewed articles, policy briefs, commentaries, case studies, and organisational reports, identified through PubMed, Google Scholar, and organisational websites, using key words and MeSH terms: ‘TB’, ‘efficiency’, ‘integration’, ‘innovation’, ‘cost-effectiveness’, ‘sustainability’, and ‘optimization’. This helped identify evidence-based practices, implementation gaps, and opportunities to improve intervention design and delivery. KIIs were conducted with six purposively selected stakeholders from international TB organisations, academic and research institutions, national TB programmes, ministries of health, and non-governmental organisations, using standardised, pre-tested questions. Inclusion criteria targeted individuals with direct roles in TB programme design, implementation, financing, or private sector engagement. Written informed consent was obtained, interviews were recorded, and transcripts were validated by participants.

The qualitative data were thematically analysed, with findings grouped into key themes to capture common patterns, divergent perspectives, and emerging insights. Results from the KIIs were triangulated with evidence from the desk review to provide a nuanced understanding of effective, scalable interventions and practical strategies to address gaps, strengthen efficiency, and optimise resources. The study was approved by the Global Fund, and written consents were obtained from participants of the KIIs.

## RESULTS

We identified scalable, cost-effective strategies to enhance TB programme efficiency and optimise resources, highlighting stakeholders’ experience and health system dynamics for sustainability. Integrating TB services within the existing programme emerged as a major driver of efficiency.

### Integrating outreach TB services

Community-based outreach models that combine contact investigation, diagnostics, treatment, and TPT in a single household visit reduce costs, improve case detection, enhance treatment adherence, and improve access to TPT. A randomised trial in Cameroon and Uganda found that integrated household contact management cost effectively reduced child TB deaths (incremental cost-effectiveness ratio <$1,000/disability-adjusted life year [DALY]).^6^ Similarly, people-centred treatment support was also cost-effective, with minimal differences in unit costs per cured patient.^7^ Community health workers’ (CHWs) engagement further improved reach and efficiency through screening, tracing, and sputum collection and provision of TPT.^8^

CHWs can simultaneously screen household members for TB disease, support TPT, identify and bring back patients who were lost to follow-up, and collect and transport sputum or other specimens from community members to health facilities for testing.

(KII – National TB Control Program [NTP], Kenya)

### Innovations and optimisation of screening and diagnosis

Combining chest X-rays (CXRs), computer-aided detection (CAD/AI), and rapid molecular tests like Xpert MTB/RIF into mobile outreach improves detection and reduces diagnostic delays, especially among high-risk, underserved populations. In Zambia, the ‘one-stop TB services’ model integrating CXR, CAD, and Xpert improved affordability and detection.^9^ In Pakistan, AI-enabled mobile chest camps with predictive mapping yielded over four cases per camp^10^ versus two in untargeted camps.^11^ In the Philippines, active case finding (ACF) using vans with CXR and Xpert MTB/RIF improved TB detection among high-risk and underserved groups.^12^

AI tools, such as CAD for X-rays, have potential for cost reduction and improved accuracy in TB screening, and diagnosis of non-TB conditions, increasing the utility of diagnostic platforms in high-burden areas.

(KII – Liverpool School of Tropical Medicine [LSTM], UK)

In high-throughput settings, CAD4TB screening costs as low as $0.25,^13^ and handheld X-rays benefit remote areas. Yet, many programmes underutilise CXR, leading to inefficiencies.

AI-powered X-ray technology offers significant potential to enhance TB screening, particularly in high-burden settings, by improving the efficiency of ACF and targeting individuals for screening.

(KII – Stop TB Partnership [STP], Switzerland)

Pairing AI-assisted X-ray screening, molecular diagnostics, and sputum pooling expanded diagnostic coverage while conserving resources. A recent modelling study in Zambia, Bangladesh, Nigeria, and Vietnam showed up to 61.5% reduction in cartridge use and a 34%–160% increase in diagnostic coverage.^14^

Utilising low-cost, rapid screening tools could ensure that diagnostic tests are reserved for people with a high pre-test probability of TB, optimising resource use.

(KII – LSTM, UK)

Sputum pooling is an efficient approach for settings with low TB positivity rates, conserving GeneXpert cartridges, and increasing testing capacity.

(KII – STP, Switzerland)

TrueNat’s extended battery life suits its use in decentralised settings.^15^ In Nigeria, stool-based TrueNat improved paediatric TB diagnosis, lowered costs, and was cost-effective.^16^ In India, Cy-TB test for TB infection was cost-effective, with bulk procurement offering further savings.^17^

### Optimising treatment and transitioning to shorter regimen

Decentralising and integrating DR-TB services have generated substantial savings per patient treated in Ethiopia ($219–$276)^18^ and India ($25,000).^19^ Shorter DR-TB and TPT regimens further improve efficiency through saving between $3,596 and $8,174 in Belarus, Georgia, Kazakhstan, and Moldova.^2^ In Brazil and South Africa, expanding access to shorter TPT regimens (e.g., 3-month Isoniazid and rifapentine – ‘3HP’) among people living with HIV substantially reduced TB cases, deaths, and DALYs.^20^ Incorporating new shorter regimens like 6-month BPaL/M (bedaquiline, pretomanid, and linezolid with/without moxifloxacin) into routine TB care was feasible and resource-efficient.^2^

Shorter DR-TB regimens such as the BPaL/M and the services can be incorporated into routine TB programmes with appropriate support to patients requiring critical care or hospitalisation.

(KII – Management Sciences for Health [MSH], Ethiopia)

### Strengthening workforce capacity through community health workers and e-learning

CHWs are central to cost-effective TB care. In Ethiopia, integrating ACF with health extension workers (HEWs – salaried CHWs) was 43.4% more cost-effective than passive case finding,^21^ and HEWs delivered community-based treatment at 39% of facility costs with similar outcomes.^22^ In Pakistan, household visits by Lady Health Workers (salaried CHWs) identified four times more TB cases at $120 per case^23^ and detected symptoms 47 days earlier than private providers.^24^ In India, TB survivors/‘TB Champions’ improved case finding and adherence, and reduced stigma, while in Vietnam, CHWs using mobile CXR boosted Xpert testing and TB notifications.^25^

Facility-based screening using X-rays could help detect more people with TB. However, there will always be a segment of the population that cannot access health care, necessitating active outreach efforts.

(KII – Global TB Partner)

Community-level strategies may need to prioritise more prevalent co-morbidities like diabetes or undernutrition depending on local epidemiological data.

(KII – LSTM, UK)

Innovations in health worker training also demonstrated potential for improved efficiency. In China, shifting from in-person to online training expanded access and reduced costs.^26^

Community-based approaches help overcome barriers such as lack of awareness, financial barriers, and stigma – TB diagnosis may lead to social exclusion.

(KII – Global TB Partner)

### Enhancing private sector engagement to expand access and improve efficiency

Innovative private sector engagement (PSE) models such as Affordable and Quality TB Tests (IPAQT), Public-Private Interface Agencies (PPIAs), and Patient-Provider Support Agency (PPSA) in India have transformed TB service delivery. IPAQT enhanced Xpert testing 10 fold and reduced prices by 30%–50%.^27^ PPIAs reached 50% of private patients at $228 per DALY.^28^ The PPSA model was adopted and scaled up through the Global Fund support and transitioned to domestic funding, ensuring sustainable PSE.^29^ In Pakistan, PSE models have expanded early diagnosis and improved case notifications and treatment adherence by integrating diverse private providers, offering patients greater flexibility.^30^

Many patients initially access health services through private providers. Engaging private providers improves access to care while leveraging existing private health care infrastructure, thereby enhancing programme efficiency.

(KII – NTP, Kenya)

The introduction of negotiated payment systems for services like GeneXpert testing in private facilities helps recover costs. Supporting private providers through capacity building, supervision, and programme linkages serves as indirect motivation.

(KII – MSH, Ethiopia)

When effectively coordinated, PSE, particularly where people first seek care outside the public sector, and community-based interventions can reduce diagnostic gaps, improve treatment coverage, and accelerate progress towards End TB targets.

Effective two-way communication between public and private providers can help reduce diagnostic delays, ultimately benefiting both patients and health systems.

(KII – LSTM, UK)

Regulatory mechanisms like Mandatory Case Notification (MCN) law can help institutionalising TB reporting by private providers, reducing reliance on incentives, ensuring compliance. However, laws alone are inadequate and require enactment, simplification including through and digitalisation of recording and reporting.

If MCN law is operationalised, there may be no need for financial incentives to encourage private providers to report cases.

(KII – Mercy Corps, Pakistan)

### Integrating TB within the broader health system and services

Integration of TB services with broader health programmes including for co-morbidities such as HIV, diabetes, and Mother and Child Health (MCH) also improves efficiency and outcomes, when tailored to local context. TB–diabetes screening in Ethiopia tripled TB yield among diabetic patients.^31^ Zimbabwe’s 3HP delivery for TPT through a fast-track HIV care model achieved 96% completion and high satisfaction.^32^ However, differentiating integration by tailoring it to the local context and disease burden was also emphasised.

While TB–HIV integration has advanced, broader integration with other health conditions requires careful balancing of individual and population-level priorities.

(KII – LSTM, UK)

Integrating specimen transport systems across TB, HIV, and other programmes reduces costs and turnaround times, and enhances traceability.^33^ In Zimbabwe, this approach cut per-sample costs by 44%, tripled annual sample volume, and reduced rejection of samples.^33^ Robust specimen transport systems in the Philippines^34^ and Pakistan^30^ reduced logistics costs and improved coordination, in resource-constrained settings.

Transporting patient samples to diagnostic facilities while allowing patients to remain in their communities increases access and reduces costs.

(KII – MSH, Ethiopia)

Establishing unified systems for sample transport ensures efficient use of existing resources, avoiding duplication across disease programmes.

(KII – NTP, Kenya)

A study in Zambia found that integrating systematic TB screening in primary health care was hindered by complex screening algorithms for children, stigma, and COVID-19, highlighting the need for local adaptation and health system strengthening.^35^

Diabetes and hepatitis C testing can also be incorporated into screening for TB at the community level to reduce the cost and duplication of services, making health care delivery more efficient.

(KII – Mercy Corps, Pakistan)

In Pakistan, TB screening among antenatal care (ANC) attendants was feasible, and important to prevent maternal and neonatal complications despite low yield.^36^ Modelling studies from Cameroon and Kenya showed that integrating TB care into child health is effective, particularly in counties with high service coverage and TB detection.^37^

In MCH clinics, health care workers are encouraged to screen children for TB during routine visits; this ensures early detection and management of TB cases among paediatric patients.

(KII – NTP, Kenya)

Immunisation campaigns, ANC visits, and polio eradication campaigns were also noted as viable touchpoints for integrating TB screening and awareness-raising activities.^38^ Table 1 distils key options and considerations to enhance efficiency and sustain TB responses in two buckets, which could be adapted to local context and resources.

**Table 1.** Summary of options to enhance efficiency and sustain and accelerate TB response.

S#	Enhancing efficiency in TB programmes	Integrating TB services with other programmes and sectors and contribute to resilient and sustainable system for health (RSSH)
1	Combine contact screening as well as TB screening in other high-risk groups with diagnosis, treatment, and provision of TPT.	Integrate disease screening (such as TB, HIV, diabetes, maternal health, and nutrition) across health programmes for comprehensive care.
2	Decentralise DR-TB activities and integrate it with DS-TB activities.	Strengthen and empower community health workers for disease detection, treatment adherence, and delivery of integrated health services across multiple conditions.
3	Promote cost-effective, shorter treatment regimens for DR-TB and TPT and children with DS-TB.	Expand and integrate sample transport networks constructed on national/local platforms and systems for TB and other diseases.
4	Optimise resources by combining screening using digital chest X-rays with AI, sputum sample pooling, and WHO-recommended rapid molecular diagnostics.	Promote multi-disease screening/testing platforms which could contribute to RSSH and pandemic preparedness and response.
5	Digitalise TB recording and reporting, enhance interoperability, and strengthen surveillance system.	Utilise digital and online platforms for training and community engagement.
6	Invest in new low-cost and more sensitive and specific tools for screening/diagnosis when available and recommended.	Leveraging digital solutions for integrated health surveillance and reporting.

DR-TB = drug-resistant TB; DS-TB = drug-sensitive TB; TPT = TB preventive treatment; AI = artificial intelligence.

## DISCUSSION

Guided by structural, institutional, and behavioural interaction concepts,^39,40^ our analysis identified five interconnected strategies – integration, optimisation, care cascade alignment, multisectoral engagement, and financing – to improve TB programme efficiency amid declining donor support and inform policy across diverse contexts.

### Integrating TB services within TB programmes

Both the literature review and KIIs highlighted that integrating TB services, combining contact investigation, diagnosis, treatment, and TPT, enhances efficiency, reduces fragmentation, and improves patient outcomes. Shorter DR-TB and TPT regimens, decentralised and integrated into routine care, enable earlier treatment, fewer visits, and higher completion, while community-based delivery supports continuity. Integrating TB and DR-TB reduces inefficiencies. Decentralised screening using digital CXR, CAD/AI, and molecular testing improves case detection, reduces delays, and increases efficiency in high-burden or underserved settings. The analysis also highlighted the value of expanding specimen transport networks to centralise diagnostic testing while maintaining community-based sample collection, reducing costs and turnaround times. Effectiveness depends on system factors: supply chains, workforce, data, and financing, and requires coordinated TB programme operations to maintain efficiency and quality.

### Leveraging innovation and optimisation of tools for screening and diagnosis

Optimising existing TB tools and technologies can enhance programme efficiency, particularly in high-burden and resource-constrained settings. The strategic integration of these tools into routine programme delivery enables earlier diagnosis, reduces operational costs, and improves overall diagnostic coverage. Evidence indicates that portable molecular platforms expand diagnostic access, decentralise services, and reduce delays, while newer screening and diagnostics tools offer affordable alternatives, when scaled through bulk procurement and appropriate training. Sputum pooling offers a cost-efficient solution in low-positivity settings (e.g., positivity rate among tested of <30%), reducing cartridge use by over half without compromising sensitivity and enabling wider testing coverage. Underutilisation of advanced tools like digital CXR and AI-enabled platforms, due to limited workforce, poor integration, and quality gaps, hinders efficiency. AI also enables multi-disease screening within a single system, improving cost-effectiveness and impact, especially in high-volume or integrated health care settings.

### Optimising algorithms and approaches along the cascade of care

Optimising diagnostic and treatment algorithms across TB care cascade, using CXR before molecular testing, and integrating AI-powered CAD, reduces confirmatory molecular testing, leading to significant cost savings and increased throughput, and expands early case detection. Community-based ACF with mobile CXR screening, and TPT provision, is more cost-effective than passive case finding, improving diagnostic access and early notifications. Transitioning from traditional classroom-based training to blended or fully online training for health care workers broadens access to continuing education, reduces travel and accommodation, and maintains workforce capacity without compromising quality.

### Integrating TB within boarder health systems and services

Evidence indicates that integrating TB services with broader health programmes, including common co-morbidities and co-conditions such as HIV, non-communicable diseases, nutrition, and MCH, improves efficiency, reduces duplication, and strengthens case finding, treatment, and care. Combined screening for TB and related conditions further enhances diagnostic yields by identifying co-morbidities earlier among high-risk groups. Integrated sample transport systems streamline multi-disease logistics, reducing turnaround times and costs while strengthening laboratory networks. AI-enabled digital CXRs enhance TB screening and detect other conditions such as chronic lung diseases, cardiovascular issues, and cancers, increasing diagnostic value. Aligning TB supervision, monitoring, and evaluation with programmes like HIV and malaria further improves efficiency by reducing duplication and consolidating resources.

### Optimising TB responses through governance, stakeholder engagement, and innovative financing

Engaging private providers, including pharmacies, general practitioners, and laboratories, expands access to affordable TB services. Innovative PSE models that incentivise private sector participation have improved case detection, treatment adherence, and programme efficiency by integrating diverse providers and tailoring interventions to local contexts. Training and supporting pharmacists and other frontline private providers with digital tools, referrals, and innovative financing, including negotiated diagnostic payment schemes, improves TB coverage and accountability, while MCN laws and effective public–private coordination reduce diagnostic delays and benefit both patients and health systems. Community-driven interventions remain a cornerstone of effective TB response, improving access, reducing diagnostic delays, and increasing patient retention. Task shifting to CHWs improves efficiency and supports continuity of care. Engaging community leaders and TB survivors reduces stigma and barriers hindering care-seeking and improves access and adherence. Combining community outreach campaigns with mobile screening and diagnostics and expanding affordable and quality TB testing in the private sector increase uptake and reduced costs. Moreover, strengthening and diversifying financing mechanisms is essential for sustainable TB programmes. Increased domestic investment enhances service continuity, reduces reliance on external aid, and supports long-term epidemic control.^41^ Social protection and health insurance schemes expand coverage, lower out-of-pocket costs, and improve outcomes by addressing socio-economic barriers. Improved coordination – such as integrating TB services into insurance programmes and supporting private-sector TB clinics – helps align resources and maximise impact. Promising innovative financing strategies include TB-specific taxes, performance-based budgeting, blended financing, and mechanisms like debt swaps and D4H arrangements. For example, Germany and Indonesia converted €75 million of debt into TB and malaria investments.^42^

Table 2 summarises opportunities to leverage additional financing for TB responses. Financial support for TB patients and their families reduces economic barriers, while performance-based financing (PBF) can enhance efficiency, programme outcomes, and resource use. Evidence from PSE in TB care shows that context-specific combinations of PBF and non-financial incentives sustain provider motivation and TB service delivery.^43^ Despite limited scope and timeline, our assessment offers practical and scalable options to sustain TB gains and strengthen patient-centred systems amid evolving funding challenges. Achieving impact requires combining efficiency with sustained investment, context-specific adaptation, and a focus on accessible, high-quality, and equitable care to all.

**Table 2.** Innovative financing strategies and approaches for consideration depending on context.

S#	Innovative financing strategies (what)	Examples (how)
1	Mobilise resources for TB programmes	•Leverage local fundraising, philanthropy, and community trust through awareness campaigns.
		•Explore community-based and social health insurance models to cover TB costs.
		•Promote inter-ministerial coordination to integrate TB services into insurance programmes.
2	Innovative funding streams	•Introduce TB-specific taxes (e.g., tobacco excise tax) and performance-based budgeting.
		•Implement district-level self-financing.
		•Blended financing – for example, together with World Bank, Asian Development Bank, African Development Bank, Islamic Bank, Gates Foundation, and Global Financing Facility.
		•Debt Swap, Debt for Health (D4H)
3	Financial assistance and performance-based payment models	•Expand financial assistance for TB patients and families through local initiatives.
		•Introducing performance-based financing to incentivise implementation of efficient interventions.

## CONCLUSION

Sustaining and accelerating TB progress requires bold leadership, domestic investments, and cross-sector partnerships scaling proven solutions. Efficiency gains must be backed by sustained funding, commitment, and focused actions including advancing research, South–South knowledge exchange, and ensuring innovations and adaptation to end TB.

## References

[1] World Health Organization. Global tuberculosis report 2025. Geneva: WHO, 2025.

[2] The Global Fund. Results report 2025. Geneva, Switzerland: The Global Fund, 2025. https://www.theglobalfund.org/en/results/.

[3] World Health Organization. United Nations general assembly high-level meeting on the fight against tuberculosis. Geneva: WHO, 2023.

[4] Pretorius C, One million lives saved per year: a cost-benefit analysis of the global plan to end tuberculosis, 2023-2030 and beyond. J Benefit Cost Anal. 2023;14(S1):337-354.

[5] Clark RA, The potential impact of reductions in international donor funding on tuberculosis in low-income and middle-income countries: a modelling study. Lancet Glob Health. 2025;13(9):e1517-e1524. 40659023 10.1016/S2214-109X(25)00232-3

[6] Brough J, Public health impact and cost-effectiveness of screening for active tuberculosis disease or infection among children in South Africa. Clin Infect Dis. 2023;77(11):1544-1551. 37542465 10.1093/cid/ciad449 PMC10686943

[7] Chavez-Rimache L, Ugarte-Gil C, Brunette MJ. The community as an active part in the implementation of interventions for the prevention and care of tuberculosis: a scoping review. PLoS Glob Public Health. 2023;3(12):e0001482. 38100540 10.1371/journal.pgph.0001482 PMC10723726

[8] Seidman G, Atun R. Does task shifting yield cost savings and improve efficiency for health systems? A systematic review of evidence from low-income and middle-income countries. Hum Resour Health. 2017;15(1):29. 28407810 10.1186/s12960-017-0200-9 PMC5390445

[9] The Global Fund. The TB quarterly update innovations contents. Geneva, Switzerland: The Global Fund, 2025. https://www.theglobalfund.org/media/uh1lxjub/tb_2025-07-quarterly-tuberculosis_update_en.pdf.

[10] EPCON. Finding TB cases in Pakistan: using AI for active case finding. Antwerp, Belgium: Epcon, 2023.

[11] Tahir A, Reviewing a model of public-private mix employed for tuberculosis control in Pakistan. Pak J Public Health. 2022;12(1):23-27.

[12] Morishita F, Bringing state-of-the-art diagnostics to vulnerable populations: the use of a mobile screening unit in active case finding for tuberculosis in Palawan, the Philippines. PLoS One. 2017;12(2):e0171310. 28152082 10.1371/journal.pone.0171310 PMC5289556

[13] Bashir S, Economic analysis of different throughput scenarios and implementation strategies of computer-aided detection software as a screening and triage test for pulmonary TB. PLoS One. 2022;17(12):e0277393. 36584194 10.1371/journal.pone.0277393 PMC9803287

[14] Codlin AJ, Expanding molecular diagnostic coverage for tuberculosis by combining computer-aided chest radiography and sputum specimen pooling: a modeling study from four high-burden countries. BMC Glob Public Health. 2024;2(1):52. 39100507 10.1186/s44263-024-00081-2 PMC11291606

[15] Treatment Action Group. TAG applauds price reduction of Molbio’s truenat diagnostics for tuberculosis. New York, NY, USA: TAG, 2023.

[16] Mafirakureva N, Evaluating the health impact, health-system costs and cost-effectiveness of using TrueNat on stool samples compared to usual care for the diagnosis of paediatric tuberculosis in primary care settings: a modelling analysis. PLoS Glob Public Health. 2025;5(6):e0004016. 40570051 10.1371/journal.pgph.0004016 PMC12200842

[17] Muniyandi M, Evaluating the cost-effectiveness of Cy-Tb for LTBI in India: a comprehensive economic modelling analysis. Int Health. 2025;17(3):259-269. 39093915 10.1093/inthealth/ihae048 PMC12045088

[18] Rosu L, Cost of treatment support for multidrug-resistant tuberculosis using patient-centred approaches in Ethiopia: a model-based method. Infect Dis Poverty. 2023;12(1):65. 37420269 10.1186/s40249-023-01116-w PMC10327382

[19] Brooks MB, Cost of inaction: a framework to estimate the economic cost of missing a patient with tuberculosis in the Indian context. BMJ Open. 2023;13(12):e070717. 10.1136/bmjopen-2022-070717 PMC10748973 38128936

[20] Nsengiyumva NP, Scaling up target regimens for tuberculosis preventive treatment in Brazil and South Africa: an analysis of costs and cost-effectiveness. PLoS Med. 2022;19(6):e1004032. 35696431 10.1371/journal.pmed.1004032 PMC9239450

[21] Woldesemayat EM. Cost-effectiveness of follow-up of chronic coughers in detecting smear-positive tuberculosis in South Ethiopia. Clin Outcomes Res. 2021;13:737-744. 10.2147/CEOR.S319588 PMC8370586 34413660

[22] Datiko DG, Lindtjørn B. Cost and cost-effectiveness of treating smear-positive tuberculosis by health extension workers in Ethiopia: an ancillary cost-effectiveness analysis of community randomized trial. PLoS One. 2010;5(2):e9158. 20174642 10.1371/journal.pone.0009158 PMC2822844

[23] Hussain H, Cost-effectiveness of household contact investigation for detection of tuberculosis in Pakistan. BMJ Open. 2021;11(10):e049658. 10.1136/bmjopen-2021-049658 PMC8543626 34686551

[24] Mercy Corps. Engagement of lady health workers leading to early diagnosis: a cross sectional study. Islamabad, Pakistan: Mercy Corps, 2018. http://www.medscape.com/viewarticle/407989.

[25] Mac TH, Optimizing active tuberculosis case finding: evaluating the impact of community referral for chest X-ray screening and Xpert testing on case notifications in two cities in Viet Nam. Trop Med Infect Dis. 2020;5(4):181. 33265972 10.3390/tropicalmed5040181 PMC7709663

[26] Wang ZY, Process evaluation of E-learning in continuing medical education: evidence from the China-gates foundation tuberculosis control program. Infect Dis Poverty. 2021;10(1):23. 33750423 10.1186/s40249-021-00810-x PMC7943261

[27] Dabas H, Initiative for promoting affordable and quality tuberculosis tests (IPAQT): a market-shaping intervention in India. BMJ Glob Health. 2019;4(6):e001539. 10.1136/bmjgh-2019-001539 PMC6936393 31908854

[28] Arinaminpathy N, Engaging with the private healthcare sector for the control of tuberculosis in India: cost and cost-effectiveness. BMJ Glob Health. 2021;6(10):e006114. 10.1136/bmjgh-2021-006114 PMC8493898 34610905

[29] Yassin MA, Leveraging Global Fund’s investments to expand innovative public-private provider engagement in TB. IJTLD Open. 2024;1(6):250-257. 39021451 10.5588/ijtldopen.24.0162 PMC11249657

[30] Rashid A, Maximizing tuberculosis services through private provider engagement – a case study from Pakistan. J Clin Tuberc Other Mycobact Dis. 2025;39:100506. 40012698 10.1016/j.jctube.2024.100506 PMC11851227

[31] Management Science for Health. Integrating service delivery for TB and diabetes Mellitus-An innovative and scalable approach in Ethiopia. New York, NY, USA: Management Sciences for Health (MSH), 2017. https://msh.org/resources/integrating-service-delivery-for-tb-and-diabetes-mellitus-an-innovative-and-scalable/.

[32] Mapingure MP, Integrating 3HP-based tuberculosis preventive treatment into Zimbabwe’s fast track HIV treatment model: experiences from a pilot study. J Int AIDS Soc. 2023;26(6):26105. 10.1002/jia2.26105 PMC10281638 37339341

[33] Muchenje W. Zimbabwe integrated sample transportation system (ISTS) case study. African Society for Laboratory Medicine 2016 International Conference, 2017. DOI: 10.13140/RG.2.2.29469.72160.

[34] Cephieid. Connecting the dots: optimizing TB diagnostics in the Philippines. Washington, DC, USA: Devex, 2024.

[35] Zulu DW, Integration of systematic screening for tuberculosis in outpatient departments of urban primary healthcare facilities in Zambia: a case study of Kitwe district. BMC Health Serv Res. 2022;22(1):732. 35655301 10.1186/s12913-022-08043-w PMC9160503

[36] Ali RF, Integrating tuberculosis screening into antenatal visits to improve tuberculosis diagnosis and care: results from a pilot project in Pakistan. Int J Infect Dis. 2021;108:391-396. 34087487 10.1016/j.ijid.2021.05.072

[37] Mafirakureva N, Cost-effectiveness of integrating paediatric tuberculosis services into child healthcare services in Africa: a modelling analysis of a cluster-randomised trial. BMJ Glob Health. 2024;9(12):e016416. 10.1136/bmjgh-2024-016416 PMC11667313 39694623

[38] Ugwu C, Integrating TB screening into house-to-house polio vaccination campaigns. Public Health Action. 2023;13(1):7-11. 10.5588/pha.22.0055 PMC10162365 37152214

[39] Umberson D, Karas Montez J. Social relationships and health: a flashpoint for health policy. J Health Soc Behav. 2010;51(Suppl):S54-S66. 20943583 10.1177/0022146510383501 PMC3150158

[40] Short SE, Mollborn S. Social determinants and health behaviors: conceptual frames and empirical advances. Curr Opin Psychol. 2015;5:78-84. 26213711 10.1016/j.copsyc.2015.05.002 PMC4511598

[41] Hallett TB, The case for optimal investment in combating HIV, tuberculosis, and malaria: a global modelling study. Lancet. 2025;406(10500):261-270. 40618766 10.1016/S0140-6736(25)00831-1

[42] The Global Fund. Innovative finances: debt swaps (Debt2Health). Geneva, Switzerland: The Global Fund, 2025. https://www.theglobalfund.org/en/how-we-raise-funds/innovative-finance/debt-swaps-debt2health/.

[43] The Global Fund. Private sector engagement in tuberculosis care. Geneva, Switzerland: The Global Fund, 2025. https://resources.theglobalfund.org/media/tslodd32/cr_private-sector-engagement-tb-care_report_en.pdf.

